# Sterility and Its Rational Treatment

**Published:** 1897-05

**Authors:** Eric Vondergoltz

**Affiliations:** New York; Professor of Gynecology, Metropolitan Post-Graduate School of Medicine


### STERILITY AND ITS RATIONAL TREATMENT.

ERIC VONDERGOLTZ, M. D., NEW YORK.

Professor of Gynecology, Metropolitan Post-Graduate School of Medicine.

In going over clinical reports the gynecologist nowadays will find that a certain number of patients will always be found under the difficult question of sterility and its treatment.

The diagnosis of sterility is very simple, but the prognosis immensely uncertain regarding cure—namely, pregnancy.

The general uncertainty of curing sterility, with all its surrounding ailments, from the standpoint of a purely surgical physician, is too well known to be reiterated. I only refer the reader to P. Mueller's (Berne, Switzerland) brilliant monograph on "Sterility: Developmental Anomalies of the Uterus," in *Cyclopedia of Obstetrics and Gynecology* (Wm.

Wood & Co.). The great difference which exists between the few results of surgical treatment and the quick cures effected by Homeopathy in my hands shows that P. Mueller's request not to overlook the constitutional treatment of the concrete case is fundamental. Under this same heading P. Mueller very clearly states that the heroic treatment will not always cure the sterility, as sometimes the curette will not reach the seat of catarrh. "But we must be prepared for many failures, for the catarrh seldom affects the uterus alone, but involves the tubes, which are not accessible to treatment."

The homeopathic treatment, or as we may call our method the constitutional treatment, reaches where the curette of the surgeon will seldom go. Only once I observed that a simple catarrhal salpingitis was gradually diminished and terminated after a very energetic curettage where the occlusion of the ostium tubae was opened and dilated by the curette.

In all my twenty-seven cases, with twenty cures (pregnancies), four absolute failures and three cases under observation, the constitutional treatment was established after the method of v. Boenninghausen, having previously looked for any anatomical reason preventing cohabitation.

Having ameliorated the case to a certain degree by the constitutional treatment (always controlled by the changes in the genital sphere), as repeated examination will reveal certain conditions, and if finally I observed that my medical treatment seemed to have reached a point where further progress was not visible, as in cases of retroflexions, the moment for action was at hand to correct the position and to observe how the corrected position would be preserved. Repeated trials and continued medical treatment finally kept the formerly and sometimes immovable retroflected uterus in the corrected position better than any pessary.

The same must be mentioned regarding endometritic catarrh. Here I followed my remedies with the greatest patience, till I could observe no further progress. If now the examination showed without any possibility of doubt that the endometritic lining of the uterus was so diseased that the removal of the endometritic mucosa would prove a benefit, I had found the right moment for action—to use the curette.

But here I must remark that while using the curette for the sake of pregnancy no antiseptic solution whatever must touch the uterine cavity, as then in my experience the certainty of result will be destroyed to at least seventy per cent. The antiseptic solutions prevent the reconstruction of an absolutely physiological normal endometritic lining—the best and safest matrix for the fecundated ovum. Only a perfectly rigid asepsis will give the fullest guarantee of success.

Incisions and excisions are unnecessary in the hands of a homeopath, as their value already has been severely questioned by the thinking old school men, as enough cervices have been observed impermeable for the finest sound, but fully permeable for the microscopical spermatozoa.

The most important question in the treatment of sterility is the result of the physical examination. And here again I must state that the ovaries and tubes be found, that the vagina and uterus must be in no atrophic condition, as absolutely necessary conditions for a good prognosis.

Other conditions, as catarrh of the tube, stenosis cervicis, pelvic inflammation, are of minor importance to the homeopathic gynecologist, so long as the constitutional treatment is instituted and kept up for a longer or shorter time, as may be necessary, depending on the experience of the treating physician.

Before I enter the most interesting cases of my record, I might refer to the Materia Medica for treatment of sterility. I cannot do this, as then I have to mention all the remedies alphabetically arranged in v. Boenninghausen's *Pocket-Book*. Certainly I did not see the generally mentioned remedies like Coffea, but saw pregnancy follow old sterilities after effected cures by either Bryonia or Palladium, or Aurum or Arnica. These remedies are (so far as I know) not mentioned as typical remedies for sterility in any book.

In the same manner as the woman is treated the husband should be the object of a constitutional treatment. The examination will reveal if there are any functional impossibilities or not.

And even in a case of absolute azoospermatism I succeeded, after having given up all hope in seeing the action of the seminal glands fully restored. In the clinical cases it will be especially shown how the constitutional treatment must comprise every detail.

Here now, as first case, the following history shall serve:

Mrs. H. A., twenty-five years old; first menses at thirteen years, always had cramps during the whole period; duration, four days; married six years; never was sick, lately suffering two months from malaria, and now for eight days from cutting pains in both ovarian regions.

History and Symptoms.—Pains in both sides, worse on pressure and moving around. Better by keeping perfectly quiet. Recently has face eruptions (pimples, itching mostly at times of the menses). Patient sweats, especially at night —the sweat has a disagreeable sour taste.

Sticking and burning pains in the urethra while passing the water—sometimes there is a feeling as if the water were passing over raw flesh.

Physical Examination.—External genitals normal. Ostium urethra somewhat swollen, mucosa protruding and inflamed, vagina narrow but very low, cervix elongated and of conical shape, uterus in sharp retroflexion, immovable and very sensitive to touch. The left ovary normal, the right one prolapsed in the Douglas, and easily palpable. The mucosa of the vagina is granular, and covered by a thick sticky mucus (reaction, sharp sour).

The examination of the patient occurred the 23d of June, 1895. At the same time I examined the husband regarding his sexual organs and semen, with absolutely positive result in regard to matrimonial life.

The remedies in the case were: Cantharis, Bryonia, etc., with more or less changing picture.

July 15th I was able to correct the retroflected position of the uterus easily, without any pain, in the genu-pectoral posture.

The leucorrhea, not yet fully cured, was diminished, and did not react sour.

The re-examination, September 23d, found the uterus in the anteverted position, and the previous symptoms of the urethra had disappeared.

Patient did not return to my office till February 12th, 1896, when engaging me for her confinement, expected July 27th, 1896.

In this case we must accept as preventing cause of pregnancy the anatomical condition of the uterus, and, further, the pathological sharp-sour reaction of the leucorrhea as immediately destroying the seminal fluid. As the patient was treated before with local applications, the condition of the vaginal mucosa and of the leucorrhea will be understood. I must mention the former treatment, as the husband narrated to me, that for the sake of family they had followed the advice of their former physician to cohabitate *a la vache* on account of the extreme retroflexion of the uterus.

The following case, No. 114 of the Metropolitan Post-Graduate Clinics, is the most interesting, perhaps, for the fact that even under the most fortunate conditions a homeopathic physician can never express a safe prognosis as to the positive or negative results—pregnancy.

Mrs. K. G., twenty-seven years old; married six years, had two children, and one abortus. Abortus three years ago, and sterile since that time from chronic pelvic inflammation subsequent to a curettage (sepsis?) after the abortion (was in bed for three months).

History and Symptoms.—Patient came under my care October 10th, 1895; she related to me that she knew the nature of her disease from her different treating physicians, especially Dr. C. Cleveland; that she had inflammation, and further that the physician of Mt. Sinai Hospital had told her that she had to undergo an operation (vaginal hysterectomy?). Said she had not been pregnant in these three years on account of the inflammation, as her other physicians had told her.

The most conspicuous symptoms were:

Patient feels as if somebody was cutting with a knife in her anus; always empty eructations; headache always moving around like cramps; pains in the ovarian regions, going down the thighs.

Physical examination showed the following anatomical condition: Uterus retroflected, painfully sensitive, immovable. The parametria on both sides a compact mass, filling out the lower pelvis. In lithotomy position the upper border of the masses could be palpated through the abdominal walls.

The diagnosis was easy—chronic pelvic inflammation.

I prescribed Palladium 30 (three doses) to be taken one every 24 hours.

October 29th.—Patient reported considerably relieved. Sac-lac.

November 2d.—Patient feels worse again. As the symptoms had not changed I gave Palladium 30 (three doses) again.

The patient did not return, and I thought that she was again under the charge of other clinics.

February 12th, 1896, patient came suddenly to my office, and most excitedly expressed her fears of being pregnant.

At my request she related that she had felt better, and therefore did not think it necessary to come, especially as she lived very far off (most clinical patients indulge in such a habit, so it is mostly by accident that the final result, cure or failure, is revealed by recommended new patients or by otherwise reported gossip).

As I examined the patient I was startled to find the following: That nearly all inflammatory swellings had disappeared, and a very well-defined pregnancy by the positive uterine signs was established.

I especially narrate this case, as the patient believed herself safe—on the authority of many physicians—not to become pregnant again.

During my whole treatment I had not thought for one moment of the possibility of seeing here a pregnancy, as the patient came for relief of her pains, and not cure of her sterility.

In this case, perhaps, on the direct inquiry I would have told the patient that there was no hope for redress of the incurred sterility.

The two related cases show the more or less passive position of the treating physician. The treatment never must be otherwise than comprising the whole case—and, finally, the treating physician must change into activity only when the critical moment will be at hand. And even the surgical cure, as demonstrated in the following cases, has only some probability of success—after a duly constitutional treatment.

Mrs. O'C., twenty-seven years old; menses at fifteen years, regular, great molimina since her eighteenth year. Had typhoid fever. Married six years. No children, neither abortus.

Symptoms.—Patient menstruates regularly every twenty-sixth day, cramp-like pains in both ovarian regions—going down to uterus, for the first two days so severe that patient stays in bed till the third day. Mostly complains of pain in her back, pains in both ovarian regions—great amelioration on bending double and gradually pressing with hands on painful spots. Death feeling in both legs and lower abdomen, and during motion in the back. The sacrum especially painful while sitting; urination, defecation normal; appetite and digestion good; general health poor; fat, flabby, and very anemic.

Patient complained especially of fluor albus.

Examination.—The external genitals well developed, vagina moderately dilated (from previous treatment). Uterus retroflected (but slightly), very sensitive, somewhat enlarged. Cervix conical, one and one-half inches long, external os closed, sound hardly could pass the canal. Both parametria so painful that a more exact examination of the ovaries was impossible from the vagina. A thick leucorrhea was covering vagina and vulva.

Patient was treated according to symptoms, with the following remedies: Colocynth, China, Calc.-carb. for seven weeks.

The re-examination then showed (after the subjective amelioration of the molimina was fully established) that the uterus could be easily handled without pain, the re-position was painless, the ovaries could be palpated, and were found normal—a slight increase of the volume of the left tube pathologically could be made out.

The leucorrhea was somewhat ameliorated and diminished in quantity. The origin of the leucorrhea was well demonstrated this time, as a thick drop could be observed hanging on the external os cervicis. The further treatment of this case was medical again—mostly pointed toward gastric symptoms, as prominent in the subjective feelings: Nux-vom., Pulsatilla, Carbo-veg., Antimon-crud.

Finally patient got impatient, and demanded, after having completed the fourth month under treatment, either to cure her of her sterility, or frankly to admit not being able to help her.

I proposed at last an operation—curetting, etc.—and as the patient and her husband were willing, two days later the patient was put under the influence of chloroform by Dr. S., and having put her in the lithotomy position, she was quickly prepared for this minor operation in the following manner: The vagina was first irrigated with five per cent. hot lysol-solution, and dried with balls of sterilized gauze for at least three minutes. The lysol-solution was removed thoroughly by an irrigation with plain but thoroughly boiled water. As the aseptic preparation of the vagina was terminated an Edebohl's speculum was inserted, and the cervix steadied by a tenaculum forceps. The cervix was then dilated by Wylie's instrument. The endometria was flushed by a stream of boiled water, and then the endometritic mucosa lining was removed thoroughly by the curette—using first large and broad sharp ones, and finally smaller ones, to be able to go into smallest crevices, and especially when possible as near as possible to the tubar ostia. Several times the work of the curette was stopped, and again a thorough irrigation of the uterus was done.

The final step was to pack loosely the uterine cavity and vagina with only steam-sterilized gauze for the next twenty-four hours.

As patient did not show any after effects, no medication was necessary. The next day the vaginal and uterine tampons were removed, and the vagina was cleaned only by irrigation with plain boiled water.

Patient was allowed to resume her household duties the fifth day after operation (September 1st, 1892).

She had her last menses April 22d, 1893—abortus July 12th, 1893, resulting from a fall (down-stairs); got pregnant again, and aborted December 4th—caused by vomiting.

Finally Mrs. O'C. was delivered (forceps) June 23d, 1894, of a boy, and the next year again, June 27th, 1895, of a boy also (self-development).

The twenty-seven cases are divided in following groups:

Pregnancies, 21; effected by medicine, 14 cases; by operation, seven cases; dubious, 2, as to the final result; no results, 4; failure by operation, 2 cases; refused operation, 2 cases.

The nine cases operated upon were treated alike. The failures in my operations were caused, perhaps, by the fact that I did not consider carefully enough the condition of the husband, as it is sometimes absolutely impossible to get the semen at all, or not at convenient times for a satisfactory examination.

In all these cases to-day I refuse any treatment, where from the first moment I notice any resistance of either side to make me fully familiar with every detail.

Finally, the following general rules could be deduced from the experience with these twenty-seven cases:

1. That the prognosis always must be given very guardedly.

2. That no result must be promised in short time.

3. That an operation will have better results the longer the patient can wait.

4. That under no circumstances must the operation be done without having treated the individual case constitutionally.

